# Anxiety, depression and relationship satisfaction in the pregnancy following stillbirth and after the birth of a live-born baby: a prospective study

**DOI:** 10.1186/s12884-018-1666-8

**Published:** 2018-01-24

**Authors:** Ida Kathrine Gravensteen, Eva-Marie Jacobsen, Per Morten Sandset, Linda Bjørk Helgadottir, Ingela Rådestad, Leiv Sandvik, Øivind Ekeberg

**Affiliations:** 1 Institute of Clinical Medicine, University of Oslo, P.O box 1171, Blindern, 0318 Oslo, Norway; 2 Department of Behavioural Sciences in Medicine, Institute of Basic Medical Sciences, University of Oslo, Oslo, Norway; 3 Department of Haematology, Oslo University Hospital, Oslo, Norway; 4 Department of Obstetrics and Gynaecology, Oslo University Hospital, Oslo, Norway; 5 Sophiahemmet University, Stockholm, Sweden; 6 Oslo Centre for Biostatistics and Epidemiology, Research support services, Oslo University Hospital, Oslo, Norway; 7 Division of Mental Health and Addiction, Oslo University Hospital, Oslo, Norway

**Keywords:** Stillbirth, Subsequent pregnancy and postpartum, Anxiety, Depression, Relationship satisfaction, The Norwegian mother and child cohort study, MoBa

## Abstract

**Background:**

Experiencing a stillbirth can be a potent stressor for psychological distress in the subsequent pregnancy and possibly after the subsequent birth. The impact on women’s relationship with her partner in the subsequent pregnancy and postpartum remains uncertain. The objectives of the study were 1) To investigate the prevalence of anxiety and depression in the pregnancy following stillbirth and assess gestational age at stillbirth and inter-pregnancy interval as individual risk factors. 2) To assess the course of anxiety, depression and satisfaction with partner relationship up to 3 years after the birth of a live-born baby following stillbirth.

**Methods:**

This study is based on data from the Norwegian Mother and Child Cohort Study, a population-based pregnancy cohort. The sample included 901 pregnant women: 174 pregnant after a stillbirth, 362 pregnant after a live birth and 365 previously nulliparous. Anxiety and depression were assessed by short-form subscales of the Hopkins Symptoms Checklist, and relationship satisfaction was assessed by the Relationship Satisfaction Scale. These outcomes were measured in the third trimester of pregnancy and 6, 18 and 36 months postpartum. Logistic regression models were applied to study the impact of previous stillbirth on depression and anxiety in the third trimester of the subsequent pregnancy and to investigate gestational age and inter-pregnancy interval as potential risk factors.

**Results:**

Women pregnant after stillbirth had a higher prevalence of anxiety (22.5%) and depression (19.7%) compared with women with a previous live birth (adjusted odds ratio (aOR) 5.47, 95% confidence interval (CI) 2.90–10.32 and aOR 1.91, 95% CI 1.11–3.27) and previously nulliparous women (aOR 4.97, 95% CI 2.68–9.24 and aOR 1.91, 95% CI 1.08–3.36). Gestational age at stillbirth (> 30 weeks) and inter-pregnancy interval <  12 months were not associated with depression and/or anxiety. Anxiety and depression decreased six to 18 months after the birth of a live-born baby, but increased again 36 months postpartum. Relationship satisfaction did not differ between groups.

**Conclusion:**

Women who have experienced stillbirth face a significantly greater risk of anxiety and depression in the subsequent pregnancy compared with women with a previous live birth and previously nulliparous women.

## Background

It is well known that a stillbirth affects women’s mental health in the short term with increased risk of anxiety, depression and posttraumatic stress [1–5]. Although psychological sequelae persist for some women [2, 6], symptoms of anxiety and depression seem to decrease within the first 1–2 years after a loss [2, 7]. A strong desire to become pregnant again is common among couples that experience perinatal loss, and about 50% embark on a new pregnancy within a year [8, 9]. The subsequent pregnancy could be regarded as an emotional stressor that may interfere with the normal grief process [10–13]. Observational studies describe elevated levels of depressive symptoms [8], posttraumatic stress symptoms [12], anxiety symptoms [8, 14] and reduced levels of prenatal attachment [14] in pregnancies subsequent to stillbirth. However, the prevalence of psychiatric disorders among women pregnant after stillbirth remains unknown. Studies are also conflicting as to whether or not the symptoms of anxiety and depression diminish after the birth of a healthy baby [8, 15, 16].

Some researchers suggest that women are more vulnerable to anxiety, depression and posttraumatic stress when a new conception occurs soon (< 12 months) after the stillbirth [8, 12]. On the other hand, the degree of grief and psychological distress may manifest itself even stronger if a woman struggles for a long time to become pregnant again [17, 18], and women pregnant after a previous loss may show less symptoms of depression compared with their non-pregnant counterparts [7]. Gestational age at the time of pregnancy loss may influence the degree of psychological distress, and grief reactions may be stronger among women with late losses [15, 19, 20]. However, third trimester losses are found to be associated with less anxiety compared with second trimester losses [7].

There has been a long-standing recognition that mental health problems like anxiety and depression, as well as marital dissatisfaction, are likely to co-occur. A woman’s relationship with her partner may be affected by pregnancy loss. While some find that the risk of subsequent partnership breakdown is increased [21, 22], others find no such association [23]. To our knowledge, there is little data on the effects of a previous stillbirth on partner relationship in the subsequent pregnancy.

Establishing the effects of a previous stillbirth on womens mental health during and after a subsequent pregnancy and identifying risk factors for anxiety and depression provides a base to improve health care guidelines. One of the main challenges when doing research in this field is the relatively low incidence of stillbirth in industrialised countries. Therefore, most studies in this field are limited by small sample sizes without adjustments for confounders or are retrospective case-control studies with imminent risks of methodological bias.

The objective of the present study was to estimate the prevalence of anxiety and depression in the subsequent pregnancy after stillbirth and to assess gestational age at the time of stillbirth and inter-pregnancy interval as individual risk factors. We also wanted to investigate the course of anxiety and depression as well as satisfaction with partner relationship from the second trimester and up to 36 months after the birth of a live-born baby.

## Methods

This study is based on selected data from the Norwegian Mother and Child Cohort Study (MoBa) and on records from the Medical Birth Registry of Norway (MBRN). MoBa is a prospective population-based pregnancy cohort study conducted by the Norwegian Institute of Public Health [24]. Participants were recruited from all over Norway from 1999 to 2008. The pregnant women consented to participate in 41% of invited pregnancies and the cohort now includes more than 95,000 women, 75,000 men and 114,000 children [25]. After registering for a routine ultrasound examination at approximately 17 weeks of gestation, all women received a postal invitation, which included an informed consent form and the first questionnaire. Follow-up is conducted by questionnaires at regular intervals. The current study is based on version VIII of the quality-assured data files released for research on 14th of February 2014 and reports data collected from 1999 to 2012.

The MBRN is based on compulsory notification of all live births, stillbirths and late miscarriages or terminations of pregnancy and includes information on current pregnancy and delivery as well as previous pregnancies [26].

This sub-study included women participating in MoBa, who were pregnant subsequent to a stillbirth, and two reference groups: 1) women with at least one live birth and no previous stillbirth and 2) nulliparous women. Women not responding to the first MoBa questionnaire or with missing MBRN data were excluded. For all three groups only women with singleton or twin pregnancies and with the MoBa pregnancy resulting in a live birth, were included. Results of the previous pregnancies were identified using data from the MoBa questionnaires and verified by information from the MBRN. Stillbirth was defined according to the World Health Organizations International Statistical Classification of Diseases 10th revision, ie, fetal death at 22 or more completed gestational weeks or birthweight > 500 g [27]. Aside from the selection criteria, the reference women were randomly selected from the entire MoBa cohort.

A previous study reported high levels of depression symptoms among 28% of women pregnant after stillbirth compared with 8% of controls [8]. Assuming a prevalence of 25% for depression or anxiety in the subsequent pregnancy after a stillbirth and 10% for reference women, a sample size of *N* = 100 in each group yields 80% power for detecting differences of this magnitude using a 5% significance level.

We identified 197 women in the MoBa cohort who had experienced stillbirth in their previous pregnancy (previous stillbirth group). The reference groups included 394 women with a live birth in their previous pregnancy (previous live birth group) and 394 nulliparous women (previously nulliparous group). We assessed data from questionnaires answered in gestational weeks 17 and 30, and 6, 18 and 36 months after the delivery of a live-born baby. Background variables from the MBRN for both the MoBa pregnancy and the previous pregnancy (previous stillbirth or live birth), were also assessed.

**Fig. 1:**
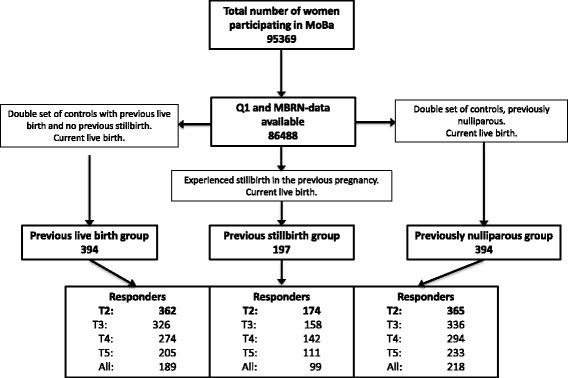
Flowchart for the selection of cases and controls. MoBa: The Norwegian; Mother and Child Cohort Study; MBRN: Medical Birth Registry of Norway; T2: Responders at 30 gestational weeks; T3: Responders at 30 gestational weeks and 6 months postpartum; T4: Responders at 30 gestational weeks and 18 months postpartum; T5: Responders at 30 gestational weeks and 36 months postpartum

At the second assessment (30 gestational weeks) 174 women with a previous stillbirth, 362 with a previous live birth and 365 nulliparous women completed the questionnaire. A flowchart for the selection of the sub-study population is provided (Fig. 1).

### Outcome measures

Depression and anxiety was measured using short versions of the Hopkins Symptom Checklist (SCL) [28] shown to correlate highly with the total score of the original scale, and to have good psychometric properties [29, 30]. We used two 4-item subscales measuring anxiety and depression during the previous 2 weeks (SCL-4a and SCL-4d). A combined score was used in pregnancy week 17. Items were scored on a Likert scale ranging from 1 (“not at all bothered”) to 4 (“very much bothered”). We defined a mean score > 1.75 on SCL-4a and/or SCL-4d as presence of anxiety and/or depression [31]. Cronbach’s alpha of internal consistency ranged from 0.69–0.80 for the anxiety subscale and 0.77–0.81 for the depression subscale.

A five-item version of the Relationship Satisfaction Scale (RS) was used to assess maternal relationship satisfaction among married/cohabiting women [32]. Developed for the MoBa study, the RS is based on core items from previously developed measures of marital satisfaction and relationship quality [33–35]. The RS correlates 0.92 with the Quality of Marriage index [36] and has a high ability to predict future break-up/divorce and life satisfaction [32, 37, 38]. The abbreviated five-item version (RS5) correlates 0.97 with the full 10-item version [32]. Each item is rated on a 6-point (1–6) Likert scale, and the total score is the mean score of all items. An average score below 4.0 implies a relatively high risk of break-up (11–15%) [32] and a score ≥ 4 was applied as cut-off to denote relationship satisfaction in this study. Cronbach’s alpha ranged from 0.87 to 0.90.

### Covariates

Sociodemographic, health related and obstetrical history factors were considered as potential confounders for the association between having experienced a stillbirth and anxiety or depression in the subsequent pregnancy. Maternal age at the time of the MoBa delivery (whole years) was retrieved from the MBRN. Co-morbidity was defined as having at least one of the following previous medical problems reported in the MBRN: Asthma, hypertension, recurrent urinary tract infections, kidney disease, rheumatoid arthritis, heart disease, epilepsy, diabetes mellitus, and/or thyroid disease. Other covariates were questionnaire data obtained at gestational week 17 or 30 and included parental status at first assessment (married/cohabiting), native language other than Norwegian, pre-pregnancy daily smoking, high pre-pregnancy body mass index (BMI ≥ 25), low education (high school or less), low income (< 200,000 Norwegian kroner/year) and previous termination(s) of pregnancy or miscarriage(s). Stressful life events were defined as having at least one of the following experiences during the last 12 months: 1) Problems at work or study place, 2) financial problems, 3) divorce/separation/relationship break-up, 4) conflicts with family or friends, 5) serious injury or illness to the woman herself or a loved one, or 6) involvement in a serious accident, fire or robbery.

### Potential predictors of anxiety and depression in the pregnancy after stillbirth

Information on gestational age at the time of stillbirth and inter-pregnancy interval was retrieved from the MBRN. Gestational age at stillbirth (based on last menstrual period and/or ultrasound measurement) was categorised as ≤ 30 weeks or > 30 weeks. Inter-pregnancy interval was defined as number of months between the date of stillbirth and the subsequent conception (estimated by ultrasound measurements) and categorised as < 12 months or ≥ 12 months.

### Statistical analyses

Categorical data were reported as proportions and compared between groups using chi-square tests. Age at the time of the MoBa delivery was reported as mean years and compared between groups using independent samples t-test. To reduce potential sample distortion caused by missing values, the Estimation-Maximation procedure in SPSS was used to impute missing values on SCL-4a, SCL-4d and RS5 if at least 50% of items were present. This resulted in 0.4% missing on SCL-4a, 0.4% missing on SCL-4d and 1.9% missing on RS5 at first assessment.

The McNemar’s test was used to analyse the differences in frequency of anxiety, depression and relationship satisfaction between different time points. Binary and multivariate logistic regression models were used to estimate odds ratios (OR) and adjusted odds ratios (aOR) for anxiety and/or depression in subsequent pregnancy among women with a previous stillbirth compared with the two reference groups. Covariates that were unevenly distributed between the groups (*p* <  0.1), associated with the outcome variable in a bivariate model (p <  0.1), and not strongly correlated (correlation coefficient <  0.7), were included in the multivariate analyses. Current age was included in all multivariate models and each final model was checked for interactions.

For the stillbirth group, separate binary regression models were used to test if gestational age at stillbirth or inter-pregnancy interval were significant predictors for anxiety or depression in the subsequent pregnancy. To preserve power and reduce the number of comparisons, we combined anxiety and depression in the subgroup analyses. Covariates and anxiety/depression in the third trimester were compared between participants completing all five questionnaires and participants who dropped out at any point after 30 gestational weeks.

All data were analysed using the Statistical Package for Social Science version 22.0 (SPSS Inc., Chicago, IL, United States). Two-sided *p*-values < 0.05 were regarded as significant.

## Results

Background characteristics are presented in Table 1. Women with a previous stillbirth and women with a previous live birth did not differ significantly according to age, but were significantly older than the previously nulliparous women. A high BMI and a low educational level was more prevalent in the previous stillbirth group compared with both reference groups. Women with a previous stillbirth more often reported stressful life events compared with women with previous live births, but not compared with previously nulliparous women.

**Table 1:** Background characteristics^a^

	Previous stillbirth *N* = 174^c^	Previous live birth *N* = 362^c^	Previously nulliparous *N* = 365^c^	*P* value	
				Stillbirth vs live birth	Stillbirth vs nulliparous
Maternal age, mean yrs. (SD)^b^	31.18 (4.63)	31.29 (4.14)	28.70 (4.45)	0.789	< 0.001
Married/cohabiting	168 (97.7)	356 (98.3)	345 (95.0)	0.595	0.152
Native language other than norwegian	8 (4.7)	16 (4.5)	23 (6.4)	0.930	0.424
Smoking^d^	32 (18.6)	46 (12.9)	70 (19.3)	0.085	0.852
BMI ≥ 25	77 (45.6)	125 (35.6)	100 (28.3)	0.029	< 0.001
Low education^e^	67 (39.0)	104 (29.1)	99 (27.9)	0.024	0.010
Low income^f^	50 (29.8)	124 (34.7)	93 (26.3)	0.259	0.414
Previous miscarriage	32 (18.4)	55 (15.2)	52 (14.2)	0.347	0.215
Previous termination of pregnancy	17 (9.8)	35 (9.7)	32 (8.8)	0.970	0.705
Co-morbidity^b^	26 (14.9)	43 (11.9)	47 (12.9)	0.321	0.512
Stressful life events	102 (58.6)	174 (48.1)	190 (52.1)	0.022	0.153
Inter-pregnancy interval^b^ < 12 months	122 (70.5)				
Gestational age at stillbirth^b^ > 30 weeks	115 (68.0)				

^a^n (%) when not other specified

^b^Data from the Norwegian Medical Birth Registry of Norway

^c^N varies due to missing data for some variables

^d^Prepregnancy daily smoking

^e^Highschool or less

^f^Maternal income < 200,000 Norwegian kroner per year

Background characteristics did not differ significantly between participants completing all five questionnaires and participants who dropped out at any point after 30 gestational weeks, with the exception of more smokers among drop-outs in the previous stillbirth group, and more participants with low education and younger age among drop-outs in the previously nulliparous group (data not shown).

### Prevalence of anxiety and depression

In the third trimester of pregnancy (30 gestational weeks), women with a previous stillbirth more often experienced anxiety (22.5%) and depression (19.7%) compared with women with previous live births (4.4% and 10.3% respectively) and previously nulliparous women (5.5% and 9.9% respectively) (Table 2).

**Table 2:** Point prevalences of anxiety, depression and relationship satisfaction

	Previous stillbirth		Previous live birth			Previously nulliparous		
	N	n (%)	N	n (%)	*P* value	N	n (%)	*P* value
Anxiety (SCL-4a > 1.75)								
*Third trimester*	173	39 (22.5)	361	16 (4.4)	< 0.001	363	20 (5.5)	< 0.001
*6 months postpartum*	158	11 (7.0)	323	15 (4.6)	0.291	335	10 (3.0)	0.041
*18 months postpartum*	140	8 (5.7)	270	17 (6.3)	0.815	291	13 (4.5)	0.573
*36 months postpartum*	108	12 (11.1)	199	5 (2.5)	0.002	229	13 (5.7)	0.076
Depression (SCL-4d > 1.75)								
*Third trimester*	173	34 (19.7)	360	37 (10.3)	0.003	364	36 (9.9)	0.002
*6 months postpartum*	158	16 (10.1)	324	28 (8.6)	0.595	334	31 (9.3)	0.766
*18 months postpartum*	140	16 (11.4)	269	41 (15.2)	0.291	291	34 (11.7)	0.938
*36 months postpartum*	108	17 (15.7)	199	16 (8.0)	0.038	229	29 (12.7)	0.443
Relationship satisfaction (RS5 ≥ 4.0)								
*Third trimester*	167	159 (95.2)	344	324 (94.2)	0.633	350	340 (97.1)	0.262
*6 months postpartum*	152	139 (91.4)	316	289 (91.5)	0.998	320	295 (92.2)	0.782
*18 months postpartum*	137	125 (91.2)	260	235 (90.4)	0.780	275	248 (90.2)	0.729
*36 months postpartum*	102	88 (86.3)	189	166 (87.8)	0.704	211	180 (85.3)	0.819

SCL-4a; Symptom Check List 4-item anxiety subscale

SCL-4d; Symptom Check List 4-item depression subscale

RS5; Relationship Satisfaction Scale, 5-item version

These differences remained significant in the multivariate analyses. The aOR for anxiety was 5.47 compared with the previous live birth group (95% CI 2.90–10.32, *p* <  0.001, adjusted for age, education, pre-pregnancy smoking and stressful life events) and 4.97 compared with the previously nulliparous group (95% CI 2.68–9.24, *p* <  0.001, adjusted for age and education). The aOR for depression was 1.91 compared with the previous live birth group (95% CI 1.11–3.27, *p* = 0.019, adjusted for age, pre-pregnancy smoking, BMI and stressful life events) and 1.91 compared with the previously nulliparous group (95% CI 1.11–3.36, *p* = 0.026, adjusted for age, education and BMI). There were no interactions present in any of the final models.

The proportion of women with both anxiety and depression in the third trimester was 12.7% among women with a previous stillbirth compared with 3.6% in each reference group (*p* <  0.001 for both comparisons).

The prevalence of anxiety and depression decreased significantly from first assessment to 6 months postpartum among women with a previous stillbirth (*p* < 0.001 for anxiety and *p* = 0.031 for depression). By six and 18 months postpartum, respectively, the prevalence of depression and anxiety was not significantly different between groups (Table 2).

**Fig. 2:**
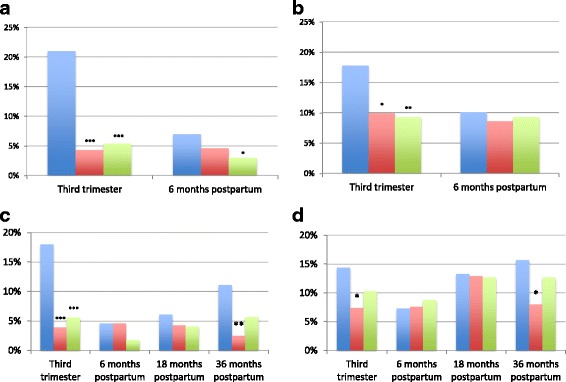
Frequency of anxiety and depression during and after pregnancy. Blue colour: Previous stillbirth group; Red colour: Previous live birth group; Green colour: Previous nulliparous group. **a** Anxiety among responders up to 6 months postpartum (158 women with previous stillbirth, 326 women with previous live births, 336 previously nulliparous women). **b** Depression among responders up to 6 months postpartum (158 women with previous stillbirth, 326 women with previous live births, 336 previously nulliparous women). **c** Anxiety among responders up to 36 months postpartum (111, women with previous stillbirth, 205 women with previous live births, 233 previously nulliparous women). **d** Depression among responders up to 36 months postpartum (111, women with previous stillbirth, 205 women with previous live births, 233 previously nulliparous women). ^*^Significant at the < 0.05 level; ^**^Significant at the < 0.01 level; ^***^Significant at the < 0.001 level

From six to 36 months postpartum, the prevalence of anxiety and depression increased significantly in the stillbirth group (*p* = 0.039 and 0.035 respectively) and the prevalence of anxiety, but not depression, increased significantly in the nulliparous group (*p *= 0.039) (Fig. 2). At 36 months postpartum, the prevalence of anxiety and depression was higher among women with a previous stillbirth compared with women with a previous live birth, but not compared with previously nulliparous women (Table 2).

The prevalence of anxiety and depression in the third trimester differed among women with a previous stillbirth completing all five questionnaires compared with drop-outs at any point after 30 gestational weeks (for anxiety 15.2 vs 32.4%, respectively, *p* = 0.007 and for depression 12.1 vs 29.7% respectively, *p* = 0.004). No such differences were observed when comparing drop-outs with responders among women with a previous live birth or previously nulliparous women.

### Inter-pregnancy interval and gestational age as risk factors for anxiety and depression in the subsequent pregnancy after stillbirth

The mean gestational age at stillbirth was 33.5 weeks (95% CI 32.5–34.6, range 20.4 to 42.6) and 115 women (68%) lost their baby at gestational age more than 30 weeks. The median number of months between stillbirth and the subsequent conception was 6 (range 1 to 183 months) and the majority of the women (*n* = 122, 70.5%) became pregnant within 12 months after the stillbirth. Inter-pregnancy interval shorter than 12 months between stillbirth and the next conception or gestational age at stillbirth > 30 weeks was not significantly associated with higher odds of anxiety and/or depression in the third trimester of the subsequent pregnancy (OR 1.49, 95% CI 0.70–3.16 *p* = 0.301 and OR 2.11, 95% CI 0.98–4.55, *p* = 0.056 respectively).

### Relationship satisfaction

The frequency of relationship satisfaction among married/cohabiting women decreased slightly in all three groups from the first assessment to 36 months postpartum (*p* = 0.012 for women with a previous stillbirth, 0.049 for women with a previous live birth, and < 0.001 for previously nulliparous women). There was no significant difference between women with a previous stillbirth and the reference groups at any point (Table 2).

## Discussion

### Main findings

Women with a previous stillbirth had higher prevalence of anxiety and depression in the subsequent pregnancy compared with women with previous live births and previously nulliparous women. The prevalence decreased considerably after the birth of a live-born baby, and was not significantly different from the reference groups by 6 months postpartum for depression, and 18 months postpartum for anxiety. However, 36 months postpartum the prevalence of anxiety and depression had increased and was again significantly higher compared with women with a previous live birth, but not compared with previously nulliparous women. Relationship satisfaction was not significantly different between groups at any time point. Having experienced a late stillbirth (> 30 weeks) or a short interval between stillbirth and the subsequent conception (< 12 months) was not significantly related to anxiety and/or depression in the subsequent pregnancy.

### Strengths and limitations

Allthough symptom levels have been studied previously, to our knowledge this is the first study to estimate the prevalence of anxiety and depression contemporaneously among women pregnant after stillbirth.

We are also the first to assess relationship satisfaction in this setting. The data is derived from a large national cohort and our sample size is larger than the majority of previous studies in this field. The prospective design of the present study minimised reporting bias and enabled a long follow-up period. Previous studies have typically made comparisons solely to a control group consisting of either women with previous live births or primigravidas. Applying two reference groups to further explore the psychological impact of stillbirth makes this study unique.

The participation rate of 40.6% at first assessment is a weakness, but as expected for population-based studies [39]. A study investigating selection bias in the MoBa study found that there was an under-representation of participants with a number of exposure variables, including previous stillbirth [40]. The same study found that prevalence estimates of exposures or outcomes may be biased due to self-selection, but that self-selection is not a problem in studies of exposure-outcome associations. We therefore argue that our findings can be generalised to other women pregnant after stillbirth. However, we cannot rule out that women with greater psychological distress after a previous stillbirth more often declined participation than women coping better after the incident. Neither can we rule out that women with mild psychological distress may have been less motivated to participate. Further, the data reported was collected over a relatively long time period (from 1999 to 2012) and changes in practice and support may have influenced our findings.

Due to ethical limitations regarding linking the MoBa data to the MBRN, the study was approved only to use a limited number of reference women instead of using the entire birth cohort as a reference. However, the prevalence of anxiety and depression among the two reference groups was similar to a control group of women without epilepsy in a previous MoBa sub-study [41].

Although the dropout rate was comparable to other studies of perinatal depression [42], missing data in the follow-up period is a concern regarding the ability of this study to make conclusions about mental health outcomes from 6 months to 3 years postpartum. As anxiety and/or depression in the subsequent pregnancy after stillbirth was more prevalent among drop-outs, anxiety and depression at follow-up is probably underestimated.

Unfortunately, we do not have reliable data regarding the prevalence of anxiety and depression before the occurrence of stillbirth. It would also be interesting to compare these women to their nonpregnant counterparts in order to assess whether the prevalence of anxiety and depression are indeed associated with being pregnant.

The estimates for anxiety and depression in our study relied on self-reporting using short-form versions of validated screening tools. Even though short-form versions affect the measurement precision, it often remains sufficient for epidemiological purposes [43]. Psychiatric symptoms may be more correctly reported in an anonymous questionnaire than in a clinical interview [44] and questionnaire-based screening tools are often used to estimate the proportion at risk of having a mental disorder in a population. However, it is important to highlight that the screening tools are not suited to make formal diagnoses.

The sample size required that data on anxiety and depression were combined in the subgroup analyses on gestational age at stillbirth and inter-pregnancy interval, limiting the generalizability of these analyses.

As we did not want to increase the risk of type II errors, adjustments for multiple comparisons were not performed and findings with *p*-values ≥ 0.01 should be considered with some caution.

### Interpretation

Our findings confirm that anxiety and depression is prevalent in the pregnancy following stillbirth. Hughes et al. [8] found that, compared with primi-gravida, women who were pregnant subsequent to a stillbirth had significantly higher levels of depression and state anxiety during pregnancy, but did not differ significantly from controls in the postpartum period and 12 months postpartum. Armstrong et al. similarily reported decreased levels of depressive symptoms and anxiety three and 8 months after the birth of a subsequently healthy infant among women with a history of perinatal death [17]. This is in accordance with our findings. However, as the follow-up period in our study extends further, we found that the prevalence of anxiety and depression increased again by 36 months post-partum. This may indicate that the subsequent birth of a live-born baby is only temporarily relieving for the psychiatric morbidity associated with stillbirth. Blackmore et al. reported that depression and anxiety associated with a previous prenatal death show a persisting pattern up to 33 months after the birth of a healthy child [18]. The latter study included mainly miscarriages, and only few stillbirths, and it is not specified whether the pregnancy is directly subsequent to the loss. It is therefore not comparable to ours.

Contrary to Hughes et al. [8], we did not find that becoming pregnant within 12 months after stillbirth significantly increases the risk of anxiety and/or depression in the subsequent pregnancy [45]. One explanation for the discrepancy may be that the majority of the women in our study conceived within a year after the loss. A Swedish study demonstrated that mothers whose baby had died in utero were given different kinds of advice concerning a suitable time for a subsequent pregnancy. The best advice seems to be that the mother should wait until she, herself, feels ready [43].

In our study, early (23–30 weeks) compared with late stillbirth (> 30 weeks) was not significantly associated with anxiety and/or depression in the subsequent pregnancy. However, the *p*-value was just slightly above the significance level. While the duration of the pregnancy could be relevant for the risk of psychological distress after a loss [19], this is probably of diminishing importance in pregnancies that have advanced beyond 22 gestational weeks.

In accordance with findings by Rådestad et al. [23], we found that a previous stillbirth did not affect satisfaction with partner relationship. Relationship satisfaction decreased slightly in all study groups and the explanation may be that having a child is by itself associated with a decline in relationship satisfaction [37, 46, 47].

## Conclusions

Anxiety and depression were more prevalent in the pregnancy following stillbirth compared with women with previous live births or previously nulliparous women. However, the prevalence declined after the birth of a live-born baby and was comparable to the reference groups by six to 18 months postpartum. After this time, depression and anxiety seemed to increase somewhat, particularly in the previous stillbirth group. Timing of the subsequent pregnancy after stillbirth was not associated with anxiety and depression in the third trimester and neither was gestational age at stillbirth. Having experienced stillbirth was not related to satisfaction with partner relationship in the subsequent pregnancy or after the birth of a live-born baby.

Implications from these findings are 1) that health care professionals in prenatal care should routinely screen for symptoms of depression and anxiety among women pregnant after stillbirth and 2) when timing a subsequent pregnancy, couples should be guided by their individual needs, taking maternal age and medical considerations into account.

Future research should evaluate the quality of care provided to reduce psychological distress in women pregnant after stillbirth. This field would also benefit from studies that take prior mental health problems into account and studies that focus on the psychological well-being of partners.

## Abbreviations

aOR: Adjusted odds ratio
BMI: Body mass index
MBRN: The Medical Birth Registry of Norway
MoBa: The Norwegian Mother and Child Cohort study
OR: Odds ratio
RS: Relationship Satisfaction Scale
RS5: Relationship Satisfaction Scale 5-item version
SCL: Hopkins Symptom Check List
SCL-4a: Hopkins Symptom Check List 4-item anxiety subscale
SCL-4d: Hopkins Symptom Check List 4-item depression subscale
